# Pneumonia and New Methicillin-resistant *Staphylococcus aureus* Clone

**DOI:** 10.3201/eid1203.051040

**Published:** 2006-03

**Authors:** Fabien Garnier, Anne Tristan, Bruno François, Jerome Etienne, Manuella Delage-Corre, Christian Martin, Nadia Liassine, Wim Wannet, François Denis, Marie-Cécile Ploy

**Affiliations:** * Laboratoire de Bactériologie-Virologie-Hygiène, Limoges, France;; † Centre National de Reference des Staphylocoques, Lyon, France;; ‡ Service de Réanimation Polyvalente, Limoges, France;; § Service d'Anatomie Pathologique, Limoges, France;; ¶ Bioanalytique-Riotton, Geneva, Switzerland;; # National Institute of Public Health and the Environment, Bilthoven, the Netherlands

**Keywords:** *Staphylococcus aureus*, methicillin-resistant, community-acquired necrotizing pneumonia, Panton-Valentin leukocidin, dispatch

## Abstract

Necrotizing pneumonia caused by *Staphylococcus aureus* strains carrying the Panton-Valentin leukocidin gene is a newly described disease entity. We report a new fatal case of necrotizing pneumonia. An *S*. *aureus* strain with an *agr*1 allele and of a new sequence type 377 was recovered, representing a new, emerging, community-acquired methicillin-resistant clone.

Necrotizing pneumonia associated with Panton-Valentine leukocidin (PVL) is a recent described clinical entity. It mainly occurs in children and young adults (median age 15 years), is fatal in 75% of cases, and is associated with a median survival time of 4 days (*1*). Necrotizing pneumonia is often preceded by a viral-like illness with signs of rhinopharyngitis. Viruses such as the influenza A virus have been isolated concomitantly with the PVL-positive *S*. *aureus* (*1*). Clinically, necrotizing pneumonia is mainly characterized by a rapidly extensive pneumonia often associated with pleural effusion and progressing towards the acute respiratory distress syndrome. Major associated signs include hemoptysis and leukopenia (<2 × 10^9^/L) (*1*). The severity of necrotizing pneumonia could be due to the products of the PVL genes, which are present in all *S*. *aureus* strains associated with this disease. The precise pathophysiology of the disease is unknown, but PVL causes tissue necrosis and leukocyte destruction. This toxin has been linked to primary skin and soft-tissue infections such as furuncles and abscesses (*1*).

Necrotizing pneumonia is mainly associated with PVL-positive methicillin-susceptible *Staphylococcus aureus* strains. Only 1 of the 16 cases reported by Gillet et al. (*1*) and 6 of the 40 cases of necrotizing pneumonia reported in the literature were associated with methicillin-resistant strains (MRSA). These PVL-positive MRSA correspond to clones that have emerged worldwide and cause community-acquired (CA) infections in patients with no risk factors. These CA-MRSA clones have a continent-specific distribution or worldwide distribution described by Vandenesch et al. (*2*). CA-MRSA clones that have spread worldwide share SCC*mec* type IV, the smallest cassette containing the *mecA* gene coding for methicillin resistance. The major European clone has a sequence type (ST) 80, as determined by multilocus sequence typing, and an *agr* (for accessory gene regulator) allele of type 3. *agr* is a global regulator controlling the expression of virulence factors in *S*. *aureus,* and has 4 alleles (*3*). PVL-positive CA-MRSA isolated in Australia have an *agr* type 3 allele and are of ST30 or ST93. The PVL-positive CA-MRSA isolated in the United States are ST1 and *agr* allele type 3, but some are ST8 or ST59 and *agr* allele type 1 (*2*). Here we report a fatal case of necrotizing pneumonia involving a 59-year-old woman infected by a new clone of PVL-positive CA-MRSA.

## Case Report

A 59-year-old woman with an unremarkable medical history was admitted to Limoges University Hospital (France) in December 2003 for acute respiratory failure. She had a 3-day history of flulike syndrome with fever, chills, cough, and myalgia and had been prescribed nonsteroidal antiinflammatory therapy. On admission, she had dyspnea, nonproductive cough, and rales in both lungs. Her arterial pressure was 60/30 mm Hg. Skin rash and diarrhea were absent. A chest radiograph showed diffuse alveolar infiltration of both lungs, without pleural effusion. Laboratory studies showed leukopenia (leukocytes = 1.66 × 10^9^/L), mild thrombocytopenia (115,000 platelets/mm^3^), hypoxemia (PaO_2_/FiO_2_ = 61), and lactic acidosis (pH = 7.29, lactates = 4.45 mmol/L). Empirical antibacterial chemotherapy combining ceftriaxone, ciprofloxacin, and gentamicin was started on admission, and mechanical ventilation was immediately necessary. Repeated tracheal aspiration was highly productive and showed hemoptysis and sustained intraalveolar plasma leakage. Despite fluid resuscitation, continuous catecholamines perfusion, dortrecogin alfa (activated) administration (Xigris, Eli Lilly and Company, Indianapolis, IN, USA), and aggressive care, her hemodynamic status deteriorated rapidly, leading to multiple organ failure and death <24 hours after admission.

Necropsy showed hemorrhagic foci in both lungs. Histopathologic studies showed total destruction of the respiratory epithelium covering the bronchi and bronchioli, together with abundant gram-positive cocci, absence of viable polymorphonuclear cells, and extensive necrosis of the alveolar septa. These lesions were characteristics of necrotizing pneumonia.

*S*. *aureus* was isolated 3 times, by culture of blood (LIM-48), lung necropsy tissue (LIM-49), and tracheal aspirate (LIM-50). The 3 isolates had the same antimicrobial drug resistance profile (resistant to oxacillin, kanamycin, tobramycin, and gentamicin) and the same pattern by pulsed-field gel electrophoresis after DNA digestion by *Sma*I (data not shown).

Influenza A virus was detected by polymerase chain reaction and immunofluorescence on the tracheal aspirate, but culture on cell lines was negative. The influenza A virus has been shown to be capable of destroying the respiratory epithelium from the trachea to the bronchioli (*1*), allowing the PVL-positive CA-MRSA to adhere to the basement membrane (*4*). PVL may diffuse locally, attracting polymorphonuclear leukocytes by its chemotactic activity and lysing them by its leukotoxicity.

Because the clinical manifestations corresponded to those of necrotizing pneumonia, isolate LIM-49 was referred (lung necropsy culture) to the French National Centre for Staphylococci. The isolate was screened for staphylococcal toxin genes, *agr* alleles, and the *mecA* gene as described elsewhere (Table) (*3**,**5**,**6*). The strain had an *agr* type 1 allele and the *lukS-PV*/*lukF-PV* genes encoding PVL (Figure), but harbored no toxin genes (*sea* through *see*, *tsst*, *eta*, and *etb* genes encoding staphylococcal enterotoxins A though E, toxic shock syndrome toxin, and exfoliative toxins A and B, respectively). The *mecA* gene was detected, together with SCC*mec* type V as described by Ito et al. (*7*). The spa type was 355 and the sequence type was ST377 (Figure). The single nucleotide difference between ST152 and ST377, which is not distinguished by using the usual primers determined by Enright et al. (*8*), was established as ST377 by using in-house primers (forward 5´-ggA CgA Agg TCA TgA TgT ATT TTT-3´ (nt 327–350), reverse 5´-CTT CTA CgC gCT CTC TTT TTA Ag-3´ (nt 564–586), according to the GenBank ID11211186 of the *gmk* gene) to amplify appropriate fragment. To our knowledge, this is the first description of such a necrotizing pneumonia–associated CA-MRSA clone. Three other ST377 strains were referred to the National Reference Centre for staphylococci in 2003. Two were isolated from patients in Europe (Netherlands and Switzerland), and 1 from an Australia patient (strain provided by G. Nimmo). The patient from the Netherlands had been treated for an abscess from which PVL-MRSA was cultured, and the Swiss patient had furunculosis. The 3 isolates harbored the SCC*mec* type V cassette, an *agr*1 allele, the same toxin gene profile, and macrorestriction pattern, *spa* type, as our isolate (Figure).

**Table:** Primers used to screen for staphylococcal toxin genes, *agr* alleles, and *mecA* gene

Gene	Primer	Oligonucleotide sequence (5´–3´)	Reference
*mecA*	*mecA*-1	AAAATCGATGGTAAAGGTTGGC	(*6*)
	*mecA*-2	AGTTCTGCAGTACCGGATTTGC	
*agr1*	*agr*1-1	ATGCACATGGTGCACATGC	(*5*)
	*agr*1-2	GTCACAAGTACTATAAGCTGCGAT	
*agr2*	*agr*2-1	ATGCACATGGTGCACATGC	(*5*)
	*agr*2-2	TATTACTAATTGAAAAGTGCCATAGC	
*agr3*	*agr*3-1	ATGCACATGGTGCACATGC	(*5*)
	*agr*3-2	GTAATGTAATAGCTTGTATAATAATACCCAG	
*agr4*	*agr*4-1	ATGCACATGGTGCACATGC	(*5*)
	*agr*4-2	CGATAATGCCGTAATACCCG	
*sea*	*sea*-1	GAAAAAAGTCTGAATTGCAGGGAACA	(*3*)
	*sea*-2	CAAATAAATCGTAATTAACCGAAGGTTC	
*seb*	*seb*-1	ATTCTAATTAAGGACACTAAGTTAGGGA	(*3*)
	*seb*-2	ATCCCGTTTCATAAGGCGAGT	
*sec*	*sec*-1	GTAAAGTTACAGGTGGCAAAACTTG	(*3*)
	*sec*-2	CATATCATACCAAAAAGTATTGCCGT	
*sed*	*sed*-1	GAATTAAGTAGTACCGCGCTAAATAATATG	(*3*)
	*sed*-2	GCTGTATTTTTCCTCCGAGAGT	
*see*	*see*-1	CAAAGAAATGCTTTAAGCAATCTTAGGC	(*3*)
	*see*-2	CACCTTACCGCCAAAGCTG	
*tsst*	*tsst*-1	TTCACTATTTGTAAAAGTGTCAGACCCACT	(*3*)
	*tsst*-2	TACTAATGAATTTTTTTATCGTAAGCCCTT	
*eta*	*eta*-1	ACTGTAGGAGCTAGTGCATTTGT	(*3*)
	*eta*-2	TGGATACTTTTGTCTATCTTTTTCATCAAC	
*etb*	*etb*-1	CAGATAAAGAGCTTTATACACACATTAC	(*3*)
	*etb*-2	AGTGAACTTATCTTTCTATTGAAAAACACTC	
LukS-PV/lukF-PV	*pvl*-1	ATCATTAGGTAAAATGTCTGGACATGATCCA	(*3*)
	*Npvl*-2	GCATCAASTGTATTGGATAGCAAAAGC	

**Figure:**
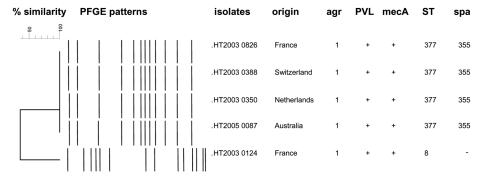
Pulsed-field gel electrophoresis (PFGE) pattern and phylogenetic tree of 4 strains ST377. *SmaI* macrorestriction patterns were digitized and analyzed by using GelCompar II (Applied Maths, Kortrijk, Belgium) to calculate Dice coefficients of correlation and to generate a dendrogram by the unweighted pair group method using arithmetic averages clustering. The scale indicates the level of pattern similarity. PVL, Panton-Valentine leukocidin; ST, sequence type.

## Conclusions

We report the identification of a new, emerging, highly virulent PVL-positive CA-MRSA clone that harbors the SCC*mec* type V cassette. This tends to confirm the continuing emergence of new CA-MRSA clones. The new clone seems to have spread rapidly in Europe, because it has been detected in patients in the Netherlands, Switzerland, and now in France. Since necrotizing pneumonia is rapidly fatal, as in the patient described here, cases of PVL-positive CA-MRSA infection must be recognized rapidly, especially in case of methicillin resistance. The therapeutic value of intravenous immunoglobulin containing anti-PVL antibodies has been reported by Gauduchon et al. (*9*) but must be confirmed.
